# Quantitative Structure–Activity Relationship Analysis of Isosteviol-Related Compounds as Activated Coagulation Factor X (FXa) Inhibitors

**DOI:** 10.3390/nu14173521

**Published:** 2022-08-26

**Authors:** Marcin Gackowski, Karolina Szewczyk-Golec, Katarzyna Mądra-Gackowska, Robert Pluskota, Marcin Koba

**Affiliations:** 1 Department of Toxicology and Bromatology, Faculty of Pharmacy, L. Rydygier Collegium Medicum in Bydgoszcz, Nicolaus Copernicus University in Torun, A. Jurasza 2 Street, PL–85089 Bydgoszcz, Poland; 2 Department of Medical Biology and Biochemistry, Faculty of Medicine, L. Rydygier Collegium Medicum in Bydgoszcz, Nicolaus Copernicus University in Torun, Karłowicza 24 Street, PL–85092 Bydgoszcz, Poland; 3 Department of Geriatrics, Faculty of Health Sciences, L. Rydygier Collegium Medicum in Bydgoszcz, Nicolaus Copernicus University in Torun, Skłodowskiej Curie 9 Street, PL–85094 Bydgoszcz, Poland

**Keywords:** isosteviol derivatives, diabetes, FXa inhibitors, multivariate adaptive regressions splines, obesity, quantitative structure–activity relationships (QSAR)

## Abstract

Stevioside, one of the natural sweeteners extracted from stevia leaves, and its derivatives are considered to have numerous beneficial pharmacological properties, including the inhibition of activated coagulation factor X (FXa). FXa-PAR signaling is a possible therapeutic target to enhance impaired metabolism and insulin resistance in obesity. Thus, the goal of the investigation was a QSAR analysis using multivariate adaptive regression splines (MARSplines) applied to a data set of 20 isosteviol derivatives bearing thiourea fragments with possible FXa inhibitory action. The best MARS submodel described a strong correlation between FXa inhibitory activity and molecular descriptors, such as: B01[C-Cl], E2m, L3v, Mor06i, RDF070i and HATS7s. Five out of six descriptors included in the model are geometrical descriptors quantifying three-dimensional aspects of molecular structure, which indicates that the molecular three-dimensional conformation is of high significance for the MARSplines modeling procedure and obviously for FXa inhibitory activity. High model performance was confirmed through an extensive validation protocol. The results of the study not only confirmed the enhancement in pharmacological activity by the presence of chlorine in a phenyl ring, but also, and primarily, may provide the basis for searching for new active isosteviol analogues, which may serve as drugs or health-beneficial food additives in patients suffering from obesity and comorbidities.

## 1. Introduction

Terpenoids belong to the largest class of structurally diverse secondary metabolites present in plants, exerting a variety of diverse pharmacological effects [1]. A widely used representative of this class of natural products, especially for its intense sweetness (about 300-times sweeter than regular sugar), is stevioside, a diterpenoid glycoside derived from *Stevia rebaudiana* Bertoni. Steviol glycosides are composed of a diterpene *ent*-kaurene core (steviol), linked to one or more glucose units. Stevia leaves have been utilized by Paraguayan Indians for centuries to sweeten Mate tea. Both stevia extract and stevioside have been known for their healthy properties by local people in South America and used in traditional medicine for centuries. Numerous studies have confirmed various therapeutic benefits of rebaudioside A (a related compound) and stevioside and its metabolic components, such as steviol and isosteviol [2,3]. These medical properties include antitumor, antidiarrheal, antihyperglycemic, antihypertensive, anti-inflammatory, diuretic and immunomodulatory activities. The aforementioned effects have driven many researchers to synthetize diverse derivatives of both stevioside and its aglycon steviol [2,4]. Stevioside has been authorized as a food additive in many countries, for instance, Japan, Korea, Brazil, the United States and the European Union [2,5]. It should be emphasized that stevioside and the products of its hydrolysis, i.e., steviol and isosteviol (ISV), are non-toxic, especially at low doses [6].

Acidic hydrolysis of stevioside affords a structural isomer of steviol, a tetracyclic diterpenoid isosteviol (ISV). Isosteviol-related compounds, possessing an *ent*-beyerane skeleton, have aroused interest because of their numerous pharmacological effects, including antibacterial, anticancer, anti-inflammatory, glucocorticoid agonist and cardioprotective properties [1,6]. Regarding examples of anticancer activity, Mizushina et al. [7] reported that ISV strongly repressed mammalian DNA polymerases and human DNA topoisomerase II. Moreover, in this study, ISV precluded the growth of human cancer cells, with LD_50_ values of 84–167 µM. In addition to this, 500 µg of ISV caused a noticeable decrease in 12-O-tetradecanoylphorbol-13-acetate (TPA)-induced inflammation (a repressive effect of 53.0%). Accordingly, Takasaki and coworkers reported that isosteviol displayed significant inhibitory activity in a two-step carcinogenesis assay, where TPA and 7,12-dimethylbenz[α]anthracene (DMBA)-induced mouse skin was used [8]. Interestingly, Al-Dhabi et al. [9] investigated in vitro antibacterial, antibiofilm, anticancer, antifungal and antioxidant properties of ISV. The studied compound showed efficacy against bacteria, such as *Staphylococcus aureus*, *Staphylococcus epidermidis* and *Klebsiella pneumoniae,* as well as against *Aspergillus niger*, *Candida albicans* and *Trichophyton mentagrophytes*. ISV also displayed reasonably better antibiofilm activity against *Escherichia coli*, *Salmonella typhi* and *Pseudomonas aeruginosa*. What is more, it showed substantial antioxidant properties and anticancer activity against Vero and MCF7 cell lines. The cardiovascular actions of isosteviol include, among other things, vasodilatation, decreasing cellular reactive oxygen species (ROS) generation and subsequent repressive effects on angiotensin-II-induced cell proliferation and endothelin-1 secretion, protective effect against the development of cardiac hypertrophy through the regulation of transient outward potassium and L-type calcium channels, improvement in H9c2 cardiomyocyte viability, restoring mitochondrial membrane potential and inhibition of cell apoptosis [6]. In addition, a study by Nordenoft et al. [10] proved antidiabetic actions of isosteviol—ISV improved glucose and insulin sensitivity in genetically obese diabetic KKay mice. What is more, it improved the lipid profile and upregulated the gene expression of key beta-cell genes, inter-alia insulin regulatory transcription factors. Thus, ISV could be considered in the prevention of obesity complications.

Obesity is a multifactorial metabolic disease characterized by complexity and chronicity (ICD-10 code E66). In recent decades, its prevalence has reached epidemic proportions, affecting almost 20% of the population worldwide [11]. This entity is mainly classified with body mass index (BMI, kg/m^2^) and adults with a BMI of 30 kg/m^2^ and above are considered to suffer from obesity. Unfortunately, obesity is viewed through the prism of the associated pathologies rather than an individual entity and, for that reason, it is often underdiagnosed or inadequately treated. The role of obesity in the development of other noncommunicable chronic diseases, such as dyslipidemia, type 2 diabetes mellitus, hypertension, cardiovascular diseases and cancer, has been well established [12]. It should be noticed that obesity is the second-most-common preventable cause of cancer. At the same time, it possibly constitutes the most common preventable cause of cancer in non-smokers [13].

The abovementioned non-caloric sweeteners used as alternatives to sucrose have attracted huge attention, not only due to a high incidence of obesity, but also diabetes and dental caries. There is constant demand for low-calorie drinks and food. They are widely used in diets for diabetics and phenylketonuria patients and diets aimed at weight loss in obese individuals. What is more, a multitude of possible biological actions of the analogues in the structure of stevioside may potentially enable the use of these compounds as drugs or food additives, playing an important role not only in weight loss, but also in reducing infectious diseases, enhancing cardiovascular protection and slowing the progress of various oxidative-stress-related diseases, diabetes and cancer. In other words, steviol-related compounds may be beneficial for individuals suffering from obesity and comorbidities.

In the present contribution, a series of isosteviol derivatives synthetized and evaluated for activated coagulation factor X (FXa) inhibitory activity by Shi et al. [14] was subjected to molecular modelling studies. In their study, Hayashi et al. [15] revealed a previously unknown role of activated coagulation factor Xa- protease-activated receptor (FXa-PAR) signaling in developing brown adipose tissue (BAT) dysfunction and systemic metabolic disorder in a murine dietary obese model. However, when an FXa inhibitor was administered, it alleviated BAT whitening, enhanced thermogenic response and systemic glucose intolerance upon dietary obesity. Moreover, ROS levels were reduced in BAT. In this light, the suppression of FXa-PAR1 signaling could become a new therapeutic target for the pharmacotherapy of obesity and diabetes. For this reason, this pilot study was conducted on twenty isosteviol derivatives bearing thiourea fragments to establish a mathematical model that may be used for the prediction of FXa inhibitory activity of new potential isosteviol derivatives beneficial for obese patients.

## 2. Materials and Methods

### 2.1. Isosteviol Analogues

Molecular modeling studies were carried out on the basis of the data on the structure of twenty novel isosteviol ((4α,8β,13β)-13–Methyl-16-oxo-17-norkauran-18-oic acid) derivatives bearing thiourea fragments and FXa inhibitory activity evaluated by Shi et al. [14]. Chemical structures and pharmacological activity are presented in Table 1.

### 2.2. Geometry Optimization and Structural Descriptors

Optimization was accomplished using semiempirical calculation with molecular mechanics (MM+) and Austin Model 1 (AM1) force fields as implemented in HyperChem 8.0 (Hypercube Inc., Gainesville, FL, USA). The geometry of each compound was smoothly optimized with the MM+ molecular mechanics method and the resulting structure became an initial structure for the AM1 semiempirical method with the application of the Polak–Ribiere algorithm to a maximum energy gradient of 0.01 kcal (Å⋅mol)^−1^. The optimization was performed for up to 30,000 steps. Calculation of molecular descriptors was performed using Dragon 7 (Talete, Milano, Italy) software. This software is able to calculate numerous molecular descriptors that are grouped into 29 logical blocks [16]. In total, over 4800 descriptors provided by Dragon 7 were subjected to a statistical analysis.

### 2.3. Statistical Analysis

The analysis is based on the following data: descriptors encoding molecular properties of a particle and the values of the negative decimal logarithm of the half-maximal inhibitory concentration (IC_50_) denoting FXa inhibitory activity, obtained from the literature data. Statistica 13.3 software (StatSoft, Cracow, Poland) was used for the purpose of the statistical analysis. Raw data comprising 4885 descriptors (acting as independent variables) and negative decimal logarithm values of the IC_50_ (pIC_50_, dependent variable) underwent a process of standardization and pre-selection. In this step, 1971 descriptors with constant and near constant values, with standard deviation less than 0.0001 and with at least one missing value were excluded. The analyses were conducted at the 5% significance level (α = 0.05). The whole set of isosteviol-related compounds was divided into a training and a test set on the basis of random sample selection in STATISTICA 13.3 Data Miner (StatSoft, Cracow, Poland). Building quantitative structure–activity relationship (QSAR) models involved applying a multivariate adaptive regression splines (MARSplines) algorithm, as implemented in STATISTICA 13.3 Data Miner. Initial evaluation of elaborated submodels led to the selection of a theoretical model suitable for predictive purposes. This assessment was performed on the basis of basic validation parameters calculated for each model (*R*^2^, *Q*^2^, *MAE*) [17], explained in Section 2.5, which provided minimal but satisfactory information about model performance.

### 2.4. MARSplines Analysis

Multivariate Adaptive Regression Splines (MARS) is an adaptive procedure for regression, capable of solving regression and classification problems. It is a relevant tool for solving high-dimensional problems, such as a large number of inputs. The non-parametric procedure requires no assumptions about the functional association between dependent and independent variables. The algorithm models a relationship with a set of coefficients and basis functions generated only from the data [18] and is also an in-built functionality of STATISTICA 13.3 Data Miner. Defined parameters in the MARSplines analysis are presented in Table 2. A detailed description of the whole procedure can be found in the study of Gackowski et al. [19], where this technique was successfully applied to predict the antitumor activity of anthrapyrazole derivatives.

### 2.5. Model Validation

The model-building process using the MARSplines procedure usually provides a portfolio of submodels differing in the maximum number of basis functions and the degree of interactions, as well as their predictive power. In the initial model validation, following indices are calculated: the determination coefficient, cross-validated determination coefficient and mean absolute error in order to choose a suitable one for the prediction of inhibition activity against FXa of isosteviol-related compounds under study [17,20].

(1) $$R^{2}=1-\frac{\sum {\left(Y_{obs}-Y_{cal}\right)}^{2}}{\sum {\left(Y_{obs}-{\bar{Y}}_{training}\right)}^{2}}$$

The coefficient of determination *R*^2^ (Equation (1)) is a measure of the variation in observed data with the predicted ones. So-called perfect correlation is noticed when *R*^2^ approaches 1. *Y_obs_* represents observed response values for the training set and *Y_calc_* represents the calculated response values for the training set of compounds. *Y_training_* is the mean observed response of the training set of compounds [17,20].

(2) $$Q^{2}\left(orQ_{LOO}^{2}\right)=1-\frac{\sum {\left(Y_{obs\left(training\right)}-Y_{pred\left(training\right)}\right)}^{2}}{\sum {\left(Y_{obs\left(training\right)}-{\bar{Y}}_{\left(training\right)}\right)}^{2}}$$

The squared leave-one-out cross-validation correlation coefficient for the modeling set (*Q*^2^) is presented in Equation (2), where *Y_obs_(training)* is the observed response and *Y_pred_ (training)* is the predicted response in the training set of compounds based on the leave-one-out (*LOO*) technique. The model is considered acceptable if *Q*^2^ exceeds 0.5 [17,20].

(3) $$MAE=\frac{\sum \left|Y_{obs}-Y_{pred}\right|}{n}$$

The mean absolute error (*MAE*) (Equation (3)) is an index for the linear relationship between predicted (*Y_pred_*) and observed (*Y_obs_*) data. It is regarded as superior to the root mean square error (RMSE) because the lack of the squared term in the formula of *MAE* provides an equal weight for all errors. Thus, *MAE* is considered a simple and more straightforward determinant of prediction errors in the context of predictive modeling studies [17,20].

The best submodel, chosen for predictive purposes on the basis of abovementioned parameters, underwent full validation procedure with the parameters as follows: *R*^2^, *Q*^2^, *Q*_F1_^2^, *Q*_F2_^2^, *Q*_F3_^2^, CCC, ∆*r_m_*^2^, $\bar{r_{m}^{2}}$, PRES, SDEP and *MAE*, which were calculated according to Roy et al. [20].

## 3. Results

More than 4800 molecular descriptors were derived for geometrically optimized structures using Dragon software, which were used as independent variables to build a model predicting the FXa inhibitory activity of twenty isosteviol analogues (for chemical structures and pharmacological activity, see Table 1).

### 3.1. Geometry Optimization

**Figure 1:**
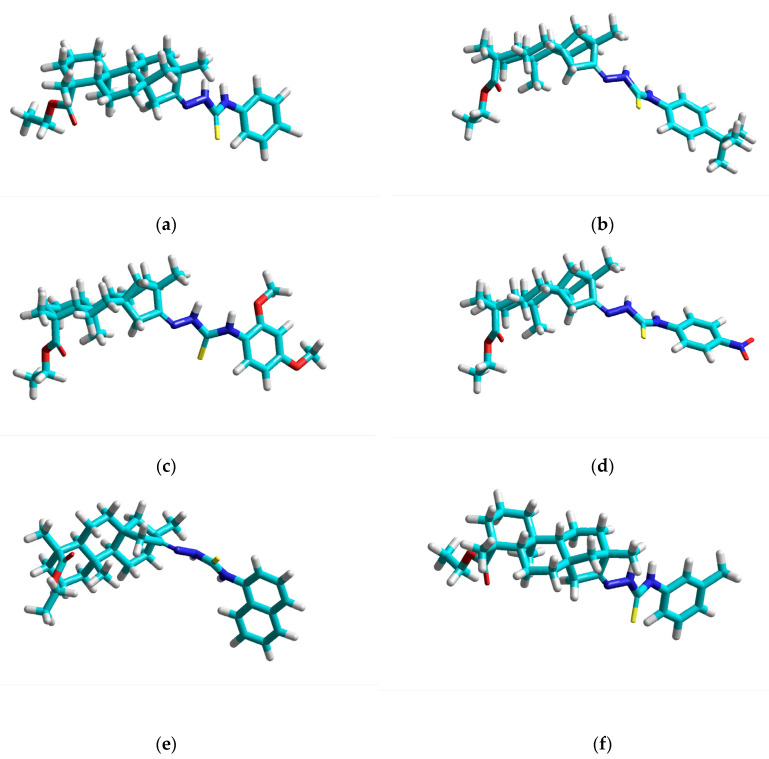
Geometrically optimized structures of selected isosteviol thiourea analogues: (**a**) i-22; (**b**) i-25; (**c**) i-29; (**d**) i-32; (**e**) i-34; (**f**) i-39.

The molecular modeling study was based on 20 isosteviol-related compounds, which in the first stage, were subjected to geometry optimization. Samples of 3D structures of studied compounds with defined geometry are presented in Figure 1.

### 3.2. Statistical Analysis

The development of the equation in the process of model building revealed a set of relevant variables, i.e., B01[C-Cl], E2m, L3v, Mor06i, RDF070i and HATS7s, which are presented in Table 3.

#### 3.2.1. Model Building and Prediction of pIC_50_ Values

The predictive quantitative structure–activity relationship model was built using the MARS algorithm. MARS built a portfolio of models using a training set of compounds, whose properties were coded as 2914 descriptors as possible predictors to predict FXa inhibitory activity denoted as pIC_50_. The degree of interaction was set to 3, which led to an incorporation of linear, second- and third-order splines into the submodels, while the maximum number of basis functions was set to 40. Finally, the best MARS submodel was chosen on the basis of validation parameters as mentioned in the Section 2.3. The predictive model comprises six descriptors, characterized in Table 4.

The quantitative structure–activity relationship MARS model is composed of quite a few interactions between molecular properties represented by descriptors as independent variables. The model begins with the constant function *B*_0_ and in subsequent steps, functions (*B_m_*) giving the best learning system fit for the current residual are added to the model according to Equation (4):

(4) $${pIC}_{50}=B_{0}+\overset{6}{\underset{m=1}{\sum }}a_{m}B_{m}$$

The elaborated MARS model contains six splines composed of single-basis functions (*B*_1_–*B*_6_). Although high-order basis functions are present in some created models, they do not appear in the predictive submodel. All basis functions (*B*_0_–*B*_6_) and their coefficients *a_m_* that form the QSAR model are presented in Table 5.

All the descriptors incorporated into the best submodel have an equal contribution, denoted as the number of appearances in a basis function (see Table 4). However, five out of six descriptors describe the molecule’s three-dimensional geometrical properties (two WHIM descriptors, 3D-MoRSE descriptor, RDF descriptor and GETAWAY descriptor) and one descriptor, i.e., a representative of 2D Atom Pairs describes the way a property is distributed along the topological structure. The block of most significance is Weighted Holistic Invariant Molecular descriptors (with a contribution of 33%), which represent a different source of chemical information [21].

#### 3.2.2. Validation and Selection of the Predictive Submodel

The MARS nonparametric procedure allowed for the establishment of a portfolio of QSAR submodels and a subsequent analysis of calculated validation parameters led to the selection of the submodel that best describes the quantitative structure–activity relationship and may be employed to predict the FXa inhibitory activity of thiourea ISV derivatives (see Table 6). The selected MARS model is characterized by the first degree of interaction and is composed of six basis functions. For this model, a perfect correlation was observed, as shown by the highest determination coefficient (*R*^2^). The squared leave-one-out cross-validation correlation coefficient exceeded the threshold of 0.5 and the lowest mean absolute error amongst all submodels was obtained (see Table 6). For the best MARS submodel, the preliminary validation was extended in order to confirm its performance. For this purpose, a full validation protocol, typical for QSAR models, was fulfilled, as described by Roy et al. [20] (see Table 7). Considering the above features along with a relatively small set of compounds submitted to the study, it should be noted that the predictive power of the obtained MARS model was relatively high.

### 3.3. Values of Predicted Data

**Figure 2:**
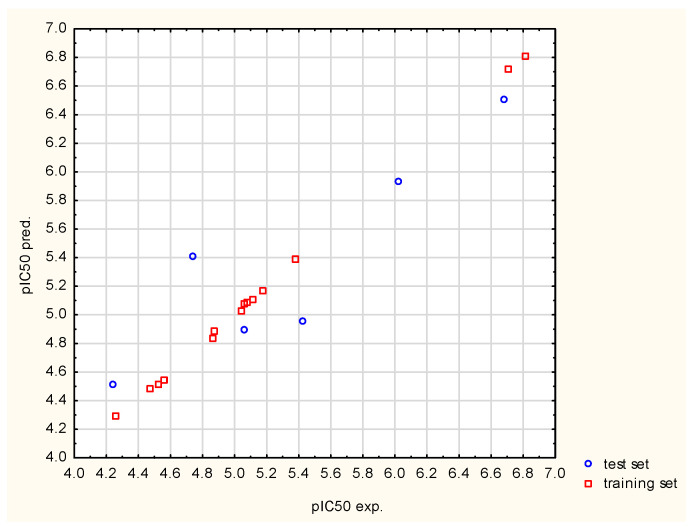
Correlation between the calculated and experimental FXa inhibitory activity of thiourea isosteviol analogues for training and test data sets. (pIC_50_-negative decimal logarithm of the half-maximal inhibitory concentration).

Values of *pIC*_50_ (${pIC}_{50calc}$) computed by the elaborated model were compared with the experimental data (${pIC}_{50exp}$) in the scatter plot, where a positive relationship is shown (see Figure 2). It can be seen that there is a greater scatter in the experimental data with respect to those determined from the model within the test set (mainly in the middle of the scatter plot) than in the case of the training set.

## 4. Discussion

Currently, obesity is a serious problem for many societies and its prevalence is increasing worldwide. It is related to a pro-coagulant state, which results in the development of numerous comorbidities, for instance, atherosclerotic disease. Coagulation factor X (FX) is involved in the coagulation cascade, which has become the main target of anticoagulant therapy. Activated FX (FXa) exerts pleiotropic biological activities, mediated through protease-activated receptor (PAR) signaling. Hayashi et al. [15] showed that coagulation factors and protease-activated receptor 1 (PAR1) are upregulated in BAT under metabolic stress. PAR1 is a prevalent form in BAT and coagulation factor-PAR1-mediated signaling contributes to a functional decline in this tissue by excessive mitochondrial production of ROS, resulting in systemic glucose intolerance in a mouse model of diet-induced obesity. According to this study, inhibition of coagulation factor-PAR1 signaling in BAT alleviates metabolic dysfunction [15]. Kaur et al. [22] investigated the involvement of the intestinal FXa-PAR2 axis in the regulation of diet-induced obesity in a murine model. Their results suggested that FXa-PAR2 signaling in the intestinal epithelium is an important factor in the regulation of postprandial glucose-dependent insulinotropic polypeptide (GIP) and early onset obesity. In view of the above observations, new therapies for the treatment of obesity and obesity-related disorders are urgently needed and new FXa inhibitors have enormous potential to be used as drugs or possible food additives.

The application of the multivariate adaptive regression splines procedure for model building in the present molecular modelling study led to the establishment of a portfolio of eight QSAR submodels (see Table 6). It should be noted that five out of eight submodels meet the initial validation requirements and may be used for the prediction of FXa inhibitory activity of isosteviol derivatives bearing thiourea fragments. The submodel that best describes the quantitative structure–activity relationship was selected for predictive purposes. Its precision, accuracy and predictability were additionally confirmed through an extended validation protocol (see Table 7). However, analysis of Figure 2 reveals that there is a greater scatter in the experimental data with respect to those determined from the model within the test than in the case of the training set. This is normal and is due to the fact that the test data were not used during the model training phase. Observed differences between experimental and calculated values are acceptable because validation parameters are within the limits described in the literature (see Table 6). Additionally, it can be noticed that the experimental and calculated values that are oscillating around the straight line represent the complete correlation. This indirectly proves that the residuals in the model values oscillate around the experimental values, which is an expected phenomenon. What attracts great attention is the simplicity of the resulting model, since it incorporates only six basis functions and first-degree interactions. This fact is strongly connected with the algorithm’s operation principle because the relationship is modeled solely on the basis of data, which, in this case, are a set of only twenty compounds [18]. Moreover, an equal contribution of variables to the model, denoted as the number of appearances in a basis function, is observed (see Table 4). In order to explore which molecular properties affect the studied activity the most, it is necessary to analyze the nature and number of individual descriptors forming the QSAR model. The following classes of descriptors may be distinguished: 2D Atom Pairs, Weighted Holistic Invariant Molecular (WHIM) descriptors, 3D-MoRSE (Molecular Representation of Structures based on Electron diffraction) descriptors, Radial Distribution Function (RDF) descriptors and GETAWAY (Geometry, Topology, and Atom-Weights Assembly) descriptors. Interestingly, five out of six predictive descriptors encode the molecule’s 3D geometrical properties (two WHIM descriptors, 3D-MoRSE descriptor, RDF descriptor and GETAWAY descriptor) and the last descriptor, i.e., a representative of 2D Atom Pairs, describes the way a property is distributed along the topological structure.

The representative of 2D Atom Pairs, i.e., B01[C-Cl], is the first to appear in the established QSAR model. This class of substructure descriptors, applicable to any pair of atoms and bond types between them, is founded on a two-dimensional representation of a molecule. Those descriptors frequently are Boolean variables encoding the presence or absence of a particular atom pair in each molecule [23]. The B01[C-Cl] descriptor included in the model is based on counting a chlorine atom in an individual compound and has a positive impact on FXa inhibitory activity. It should be noted that the reported phenomenon is consistent with the results of a study conducted by Shi et al. [14], in which the positive impact of the introduction of a chlorine atom on the inhibitory activity of studied compounds was also emphasized. The introduction of electron-donating groups into the phenyl ring had a negative influence on FXa inhibitory activity, but the introduction of the chlorine atom had the exact opposite effect.

Weighted holistic invariant molecular descriptors contain global and directional information and are estimated by a principal component analysis on Cartesian coordinates of the atoms weighted in different ways. They encode relevant molecular 3-dimensional information concerning molecular size, shape, symmetry, and atom distribution with respect to invariant reference frames [21]. There are two types of WHIM descriptors (E2m and L3v) incorporated into the obtained model, with their largest cumulative contribution as about 33%. L3v reflects the size of the molecule, whereas E2m is a variable encoding atomic distribution.

3D-MoRSE (Molecular Representation of Structures based on Electron diffraction) descriptors were designed for encoding the 3D structure of a molecule by a fixed number of variables. Despite the fact that descriptors from this block comprise information pertaining to the whole molecule, they are defined mainly by short-distance atom pairs [24]. Descriptor Mor06i is a signal 06/weighted by ionization potential, which, in the case of the studied compounds, may increase contributions of chlorine.

Radial distribution function descriptors with RDF070i, included in the elaborated model, are based on the distance distribution in the geometrical representation of a molecule and constitute a radial distribution function code, which exhibits similar characteristics as the 3D-MoRSE code. In addition to information about interatomic distances of a whole molecule, they contain information on bond distances, ring types, planar and nonplanar systems and atom types. RDF descriptors are unique, concerning the three-dimensional arrangement of the atoms, invariable against the translation and rotation of the entire molecule and independent of the number of atoms [25,26]. In this study, the presence of the RDF070i descriptor in the QSAR model suggests a certain dependence between FXa inhibitory activity of thiourea isosteviol analogues and the 3D distribution of ionization potential.

The last variable belongs to GETAWAY descriptors. This block of descriptors has been proposed in order to match 3D-molecular geometry, atom relatedness and chemical information with the use of various atomic weighting schemes. These descriptors are a source of local or/and distributed information on molecular structure [27]. The HATS7s descriptor, which is included in the resulting model, is calculated from the leverage matrix, obtained by the centered atomic coordinates and related to intrinsic properties of an individual molecule.

To summarize, the accomplished model contains one 2D and five 3D descriptors, which suggests that the molecular 3D conformation is very important for the MARSplines modeling process and, as a consequence, for FXa inhibitory activity. In addition, what should be emphasized is the joint use of GETAWAY and WHIM descriptors, with a cumulative share in the model of 50%. This combination of variables provides more predictive models, especially in the case of biological activities, as suggested by Consonni et al. [27]. The elaborated model has a very high application value, confirmed by an extensive validation protocol and, for that reason, it may be employed to predict FXa inhibitory activity of new isosteviol analogues bearing thiourea fragments. In light of this fact, the MARSplines procedure presented in this study may become either a part of a computer-aided drug design or a QSAR strategy for searching new health-beneficial food additives.

## 5. Conclusions

A set of isosteviol thiourea derivatives was subjected to a molecular modeling study and an approach of MARSplines was employed for predicting FXa inhibitory activity. The developed QSAR model reveals information about the importance of the presence of chlorine atoms (B01[C-Cl]), the uniform distribution of the atomic mass (E2m), the molecular volume (L3v), the 3D molecular distribution of ionization potential (Mor06i and RDF070i) and the intrinsic properties of a molecule (HATS7s). Five out of six descriptors are geometrical descriptors quantifying three-dimensional aspects of molecular structure. Despite a relatively small set of studied compounds, the high application value of the obtained model was confirmed through an extensive validation protocol typical of QSAR models. Consequently, all calculated validation coefficients reflect the predictive power of regression. As FXa-PAR signaling is a possible therapeutic target to enhance impaired metabolism and insulin resistance in obesity, the predictive model may represent a valuable tool in searching for new active isosteviol analogues. Finally, the results of the present study confirmed an enhancement in pharmacological activity of isosteviol analogues by the presence of chlorine in the phenyl ring. Nevertheless, future studies are necessary to investigate the influence of a wider variety of substituents.

## Figures and Tables

**Table 1:** Chemical structures and FXa inhibitory activity of isosteviol derivatives studied.

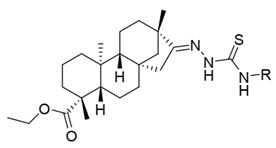			
**Compound**	**Set**	R *	Inhibition Activity Against FXa **: IC_50_ ***, M ****
i20	training	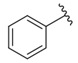	13,382.4 ± 183.85
i21	test	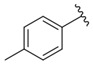	57,733.6 ± 315.07
i22	training	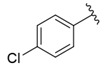	152.78 ± 3.18
i23	training	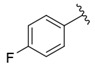	27,546.6 ± 391.27
i24	training	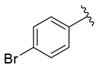	8772.8 ± 25.43
i25	training	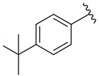	9034.4 ± 16.58
i26	training	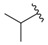	54,893.0 ± 588.77
i27	test	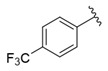	8658.8 ± 40.20
i28	training	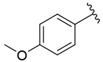	6651.8 ± 40.00
i29	training	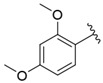	33,578.2 ± 275.65
i30	test	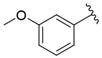	18,409.0 ± 435.88
i31	training	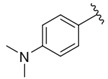	29,616.6 ± 349.80
i32	training	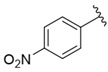	8463.4 ± 21.64
i33	training	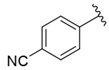	13,591.6 ± 410.15
i34	test	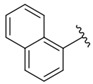	955.12 ± 18.74
i35	training	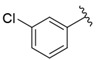	196.34 ± 5.37
i36	training	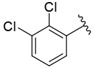	7727.8 ± 11.63
i37	training	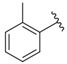	4154.2 ± 50.22
i38	test	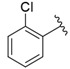	209.38 ± 4.76
i39	test	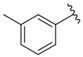	3759.2 ± 28.12

* a side chain of a particular isosteviol thiourea analogue; ** activated coagulation factor X; *** the half-maximal inhibitory concentration; **** molarity.

**Table 2:** User-defined parameters of MARSplines procedure.

Options	Values
Maximum number of basis functions	40
Degree of interactions	3
Penalty	2
Threshold	0.0005
Apply pruning	YES

**Table 3:** Values of significant molecular descriptors for the tested isosteviol derivatives.

Compound	Set	Descriptors					
		B01[C-Cl] *	E2m **	L3v ***	Mor06i ****	RDF070i *****	HATS7s ******
i20	training	0	0.166	1.551	−4.428	50.027	0.614
i21	test	0	0.164	1.477	−4.302	55.231	0.592
i22	training	1	0.139	1.525	−3.421	50.427	0.677
i23	training	0	0.153	1.518	−3.909	51.834	0.757
i24	training	0	0.114	1.519	−3.619	50.99	0.650
i25	training	0	0.155	1.54	−4.578	54.079	0.477
i26	training	0	0.183	1.468	−4.294	54.897	0.585
i27	test	0	0.133	1.485	−4.321	53.827	0.645
i28	training	0	0.151	1.62	−4.445	52.251	0.577
i29	training	0	0.194	1.444	−4.451	47.955	0.559
i30	test	0	0.152	1.685	−4.568	49.918	0.600
i31	training	0	0.157	1.469	−4.434	59.622	0.525
i32	training	0	0.134	1.577	−3.925	50.96	0.633
i33	training	0	0.148	1.495	−4.196	50.649	0.646
i34	test	0	0.178	2.086	−5.578	52.789	0.566
i35	training	1	0.147	1.574	−3.515	54.923	0.629
i36	training	1	0.281	1.716	−1.044	44.579	0.597
i37	training	0	0.161	1.824	−4.695	57.552	0.546
i38	test	1	0.191	1.296	−4.037	45.968	0.597
i39	test	0	0.165	1.648	−4.638	55.429	0.609

* Presence/absence of C-Cl at topological distance 1; ** 2nd component accessibility directional WHIM index/weighted by mass; *** 3rd component size directional WHIM index/weighted by van der Waals volume; **** signal 06/weighted by ionization potential; ***** Radial Distribution Function—070/weighted by ionization potential; ****** leverage-weighted autocorrelation of lag 7/weighted by I-state.

**Table 4:** Selected descriptors and the number of their appearances in the basis functions of the MARS model.

Symbol	Definition	Block	Dimensionality	Number in the Basis Function
B01[C-Cl]	Presence/absence of C-Cl at topological distance 1	2D Atom Pairs	2D	1
E2m	2nd component accessibility directional WHIM index/weighted by mass	WHIM * descriptors	3D	1
L3v	3rd component size directional WHIM index/weighted by van der Waals volume	WHIM descriptors	3D	1
Mor06i	signal 06/weighted by ionization potential	3D-MoRSE ** descriptors	3D	1
RDF070i	Radial Distribution Function—070/weighted by ionization potential	RDF *** descriptors	3D	1
HATS7s	leverage-weighted autocorrelation of lag 7/weighted by I-state	GETAWAY **** descriptors	3D	1

* Weighted Holistic Invariant Molecular descriptors; ** Molecular Representation of Structures based on Electron diffraction; *** Radial Distribution Function; **** Geometry, Topology and Atom-Weights Assembly.

**Table 5:** The basis spline functions.

*B_m_* *	Definition	*a_m_* **
*B*_0_	1	5.74300
*B*_1_	(B01[C-Cl])_+_	2.08922
*B*_2_	(E2m-0.11400)_+_	−1.17409
*B*_3_	(L3v-1.44400)_+_	2.16641
*B*_4_	(Mor06i+4.69500)_+_	−3.32023
*B*_5_	(RDF070i-44.57900)_+_	−4.22030
*B*_6_	(HATS7s-0.47700)_+_	−1.17824

* basis function ** coefficient of a basis function.

**Table 6:** Values of validation parameters of models obtained with the MARSplines procedure (the optimal model marked in blue).

Degree of Interaction	Number of Basis Functions	*R* ^2^	*Q* ^2^	*MAE*
1	2	0.99272	0.15208	0.2973
	6	0.99846	0.79223	0.1017
2	1	0.98984	0.17375	0.4235
	5	0.99745	0.67195	0.1635
	12	0.99631	0.50117	0.1476
3	1	0.98984	0.17375	0.4235
	5	0.99751	0.67195	0.1635
	12	0.99747	0.65840	0.1313

**Table 7:** Values of validation parameters of the best MARS submodel.

Parameter	Value	Threshold	Meaning [20]
$R^{2}=1-\frac{\sum {\left(Y_{obs}-Y_{cal}\right)}^{2}}{\sum {\left(Y_{obs}-{\bar{Y}}_{training}\right)}^{2}}$	0.9985	~1	a measure of the variation of observed with the predicted data
$Q^{2}\left(orQ_{LOO}^{2}\right)=1-\frac{\sum {\left(Y_{obs\left(training\right)}-Y_{pred\left(training\right)}\right)}^{2}}{\sum {\left(Y_{obs\left(training\right)}-{\bar{Y}}_{\left(training\right)}\right)}^{2}}$	0.7922	≥0.5	cross-validated *R*^2^ (*Q*^2^) tested for internal validation
$Q_{F1}^{2}=1-\frac{\sum {\left(Y_{obs\left(test\right)}-Y_{pred\left(test\right)}\right)}^{2}}{\sum {\left(Y_{obs\left(test\right)}-{\bar{Y}}_{\left(training\right)}\right)}^{2}}$	0.9874	≥0.5	it measures the correlation between the observed and predicted data of the test set
$Q_{F2}^{2}=1-\frac{\sum {\left(Y_{obs\left(test\right)}-Y_{pred\left(test\right)}\right)}^{2}}{\sum {\left(Y_{obs\left(test\right)}-{\bar{Y}}_{\left(test\right)}\right)}^{2}}$	0.7927	≥0.5	almost equal or closer values of Q^2^_(F2)_ and Q^2^_(F1)_ infer that the training set mean lies in the close propinquity to that of the test set
$Q_{F3}^{2}=1-\frac{[\sum {\left(Y_{obs\left(test\right)}-Y_{pred\left(test\right)}\right)}^{2}]/n_{test}}{\left[\sum {\left(Y_{obs\left(train\right)}-{\bar{Y}}_{\left(train\right)}\right)}^{2}\right]/n_{train}}\;$	0.9706	≥0.5	it is a measure of the model predictability
$CCC=\frac{2\sum_{i=1}^{n}\left(x_{i}-\bar{x}\right)\left(y_{i}-\bar{y}\right)}{\sum_{i=1}^{n}{\left(x_{i}-\bar{x}\right)}^{2}+\sum_{i=1}^{n}\left(y_{i}-\bar{y}\right)+n\left(\bar{x}-\bar{y}\right)}$	0.9635	~1	concordance correlation coefficient (CCC) measures both precision and accuracy, detecting the distance of the observations from the fitting line and the degree of deviation of the regression line from that passing through the origin, respectively
$\bar{r_{m}^{2}}=\frac{\left(r_{m}^{2}+{r^{\prime }}_{m}^{2}\right)}{2}$$\;\mathrm{and}\;∆r_{m}^{2}=\left|r_{m}^{2}-r\prime_{m}^{2}\right|,$ $\mathrm{where}\;r_{m}^{2}=r_{}^{2}\times \left(1-\sqrt{r_{}^{2}-}r_{0}^{2}\right)$ ${r^{\prime }}_{m}^{2}=r_{}^{2}\times \left(1-\sqrt{r_{}^{2}-}{r^{\prime }}_{0}^{2}\right)$ $\mathrm{And}\;\mathrm{parameters}\;r^{2}\;\mathrm{and}\;r_{0}^{2}\;\mathrm{are}\;\mathrm{denoted}\;\mathrm{as}\;\mathrm{follows}:$ $r_{0}^{2}=1-\frac{\sum {\left(Y_{obs}-k\times Y_{pred}\right)}^{2}}{\sum {\left(Y_{obs}-{\bar{Y}}_{obs}\right)}^{2}}\;\mathrm{and}$ $r\prime_{0}^{2}=1-\frac{\sum {\left(Y_{pred}-k\prime \times Y_{obs}\right)}^{2}}{\sum {\left(Y_{pred}-{\bar{Y}}_{pred}\right)}^{2}}$ The terms k and k′ are explained as follows: $k=\frac{\sum {\left(Y_{obs}\times Y_{pred}\right)}^{}}{\sum {\left(Y_{pred}\right)}^{2}}$ $\;\mathrm{and}\;k^{\prime }=\frac{\sum {\left(Y_{obs}\times Y_{pred}\right)}^{}}{\sum {\left(Y_{obs}\right)}^{2}}$	0.0196 and 0.9216	$∆r_{m}^{2}$$\;<\;0.2\;\mathrm{provided}\;\mathrm{that}\;\mathrm{the}\;\mathrm{value}\;\mathrm{of}\;\bar{r_{m}^{2}}$ 2 > 0.5	they reflect the overall predictability of the model for the whole data set
$PRESS=\sum {\left(Y_{obs}-Y_{pred}\right)}^{2}$	0.8154		assesses the model using the predicted residual sum of squares
$SDEP=\sqrt{\frac{\mathrm{PRESS}}{n}}$	0.2020		standard deviation of error of prediction (SDEP) is calculated from PRESS
$MAE=\frac{\sum \left|Y_{obs}-Y_{pred}\right|}{n}$	0.1017		index of errors in the context of predictive modeling studies

## Data Availability

Not applicable.

## References

[1] Ozsvár D., Nagy V., Zupkó I., Szakonyi Z. (2021). Synthesis and Biological Application of Isosteviol-based 1,3-aminoalcohols. Int. J. Mol. Sci..

[2] Chatsudthipong V., Muanprasat C. (2009). Stevioside and Related Compounds: Therapeutic Benefits beyond Sweetness. Pharmacol. Ther..

[3] Hanson J.R., De Oliveira B.H. (1993). Stevioside and Related Sweet Diterpenoid Glycosides. Nat. Prod. Rep..

[4] Moons N., De Borggraeve W., Dehaen W. (2012). Stevioside and Steviol as Starting Materials in Organic Synthesis. Curr. Org. Chem..

[5] Corporate Authors (2012). EC Regulation No. 231/2012, C.R. Commission Regulation (EU) No 231/2012. Off. J. Eur. Union.

[6] Wang M., Li H., Xu F., Gao X., Li J., Xu S., Zhang D., Wu X., Xu J., Hua H. (2018). Diterpenoid Lead Stevioside and Its Hydrolysis Products Steviol and Isosteviol: Biological Activity and Structural Modification. Eur. J. Med. Chem..

[7] Mizushina Y., Akihisa T., Ukiya M., Hamasaki Y., Murakami-Nakai C., Kuriyama I., Takeuchi T., Sugawara F., Yoshida H. (2005). Structural Analysis of Isosteviol and Related Compounds as DNA Polymerase and DNA Topoisomerase Inhibitors. Life Sci..

[8] Takasaki M., Konoshima T., Kozuka M., Tokuda H., Takayasu J., Nishino H., Miyakoshi M., Mizutani K., Lee K.H. (2009). Cancer Preventive Agents. Part 8: Chemopreventive Effects of Stevioside and Related Compounds. Bioorg. Med. Chem..

[9] Abdullah Al-Dhabi N., Valan Arasu M., Rejiniemon T.S. (2015). In Vitro Antibacterial, Antifungal, Antibiofilm, Antioxidant, and Anticancer Properties of Isosteviol Isolated from Endangered Medicinal Plant Pittosporum Tetraspermum. Evid.-Based Complement. Altern. Med..

[10] Nordentoft I., Jeppesen P.B., Hong J., Abudula R., Hermansen K. (2008). Isosteviol Increases Insulin Sensitivity and Changes Gene Expression of Key Insulin Regulatory Genes and Transcription Factors in Islets of the Diabetic KKAy Mouse. Diabetes Obes. Metab..

[11] Yumuk V., Tsigos C., Fried M., Schindler K., Busetto L., Micic D., Toplak H. (2015). European Guidelines for Obesity Management in Adults. Obes. Facts.

[12] Carretero Gómez J., Ena J., Arévalo Lorido J.C., Seguí Ripoll J.M., Carrasco-Sánchez F.J., Gómez-Huelgas R., Pérez Soto M.I., Delgado Lista J., Pérez Martínez P. (2021). Obesity Is a Chronic Disease. Positioning Statement of the Diabetes, Obesity and Nutrition Workgroup of the Spanish Society of Internal Medicine (SEMI) for an Approach Centred on Individuals with Obesity. Rev. Clínica Española.

[13] Lazarus E., Bays H.E. (2022). Cancer and Obesity: An Obesity Medicine Association (OMA) Clinical Practice Statement (CPS). Obes. Pillars.

[14] Shi Y., Pan B.W., Li W.C., Wang Q., Wu Q., Pan M., Fu H.Z. (2020). Synthesis and Biological Evaluation of Isosteviol Derivatives as FXa Inhibitors. Bioorg. Med. Chem. Lett..

[15] Hayashi Y., Shimizu I., Yoshida Y., Ikegami R., Suda M., Katsuumi G., Fujiki S., Ozaki K., Abe M., Sakimura K. (2022). Coagulation Factors Promote Brown Adipose Tissue Dysfunction and Abnormal Systemic Metabolism in Obesity. iScience.

[16] Talete SRL List of Molecular Descriptors Calculated by Dragon. http://www.talete.mi.it/products/dragon_molecular_descriptor_list.pdf.

[17] Tropsha A. (2010). Best Practices for QSAR Model Development, Validation, and Exploitation. Mol. Inform..

[18] Friedman J.H. (1991). Multivariate Adaptive Regression Splines. Ann. Stat..

[19] Gackowski M., Szewczyk-Golec K., Pluskota R., Koba M., Madra-Gackowska K., Woźniak A. (2022). Application of Multivariate Adaptive Regression Splines (MARSplines) for Predicting Antitumor Activity of Anthrapyrazole Derivatives. Int. J. Mol. Sci..

[20] Roy K., Ambure P., Kar S., Ojha P.K. (2018). Is It Possible to Improve the Quality of Predictions from an “Intelligent” Use of Multiple QSAR/QSPR/QSTR Models?. J. Chemom..

[21] Todeschini R., Gramatica P. (1997). The Whim Theory: New 3D Molecular Descriptors for Qsar in Environmental Modelling. SAR QSAR Environ. Res..

[22] Kaur D., Thati M., Ruf W. Factor X—Protease Activated Receptor-2 Signaling in the Regulation of Diet-Induced Obesity. https://abstracts.isth.org/abstract/factor-x-protease-activated-receptor-2-signaling-in-the-regulation-of-diet-induced-obesity.

[23] Cao D.S., Liang Y.Z., Xu Q. (2012). Molecular Descriptors Guide Description of the Molecular Descriptors Appearing in the ChemoPy Software Package © 2012 China Computational Biology Drug Design Group Table of Contents. Man. Chemopy.

[24] Devinyak O., Havrylyuk D., Lesyk R. (2014). 3D-MoRSE Descriptors Explained. J. Mol. Graph. Model..

[25] Todeschini R., Consonni V. (2000). Handbook of Molecular Descriptors.

[26] Suleiman M., Klenina O.V., Ogurtsov V. (2014). Synthesis, Biological Activity Evaluation and QSAR Studies of Novel 3- Synthesis, Biological Activity Evaluation and QSAR Studies of Novel. J. Chem. Pharm. Res..

[27] Consonni V., Todeschini R., Pavan M., Gramatica P. (2002). Structure/Response Correlations and Similarity/Diversity Analysis by GETAWAY Descriptors. 2 Application of the Novel 3D Molecular Descriptors to QSAR/QSPR Studies. J. Chem. Inf. Comput. Sci..

